# Robustness of magnetic resonance radiomic features to pixel size resampling and interpolation in patients with cervical cancer

**DOI:** 10.1186/s40644-021-00388-5

**Published:** 2021-02-02

**Authors:** Shin-Hyung Park, Hyejin Lim, Bong Kyung Bae, Myong Hun Hahm, Gun Oh Chong, Shin Young Jeong, Jae-Chul Kim

**Affiliations:** 1 Department of Radiation Oncology, School of Medicine, Kyungpook National University Hospital, 130 Dongduk-Ro, Jung-Gu, Daegu, 41944 Republic of Korea; 2 Cardiovascular Research Institute, School of Medicine, Kyungpook National University, Daegu, Republic of Korea; 3 Department of Radiology, School of Medicine, Kyungpook National University, Daegu, Republic of Korea; 4 Department of Obstetrics and Gynecology, School of Medicine, Kyungpook National University, Daegu, Republic of Korea; 5 Department of Obstetrics and Gynecology, Kyungpook National University Chilgok Hospital, Daegu, Republic of Korea; 6 Clinical Omics Research Center, School of Medicine, Kyungpook National University, Daegu, Republic of Korea; 7 Department of Nuclear Medicine, School of Medicine, Kyungpook National University, Daegu, Republic of Korea

**Keywords:** Radiomics, Cervical cancer, Magnetic resonance imaging, Pixel size resampling, Interpolation, Robustness

## Abstract

**Background:**

Radiomics is a promising field in oncology imaging. However, the implementation of radiomics clinically has been limited because its robustness remains unclear. Previous CT and PET studies suggested that radiomic features were sensitive to variations in pixel size and slice thickness of the images. The purpose of this study was to assess robustness of magnetic resonance (MR) radiomic features to pixel size resampling and interpolation in patients with cervical cancer.

**Methods:**

This retrospective study included 254 patients with a pathological diagnosis of cervical cancer stages IB to IVA who received definitive chemoradiation at our institution between January 2006 and June 2020. Pretreatment MR scans were analyzed. Each region of cervical cancer was segmented on the axial gadolinium-enhanced T1- and T2-weighted images; 107 radiomic features were extracted. MR scans were interpolated and resampled using various slice thicknesses and pixel spaces. Intraclass correlation coefficients (ICCs) were calculated between the original images and images that underwent pixel size resampling (OP), interpolation (OI), or pixel size resampling and interpolation (OP+I) as well as among processed image sets with various pixel spaces (P), various slice thicknesses (I), and both (P + I).

**Results:**

After feature standardization, ≥86.0% of features showed good robustness when compared between the original and processed images (OP, OI, and OP+I) and ≥ 88.8% of features showed good robustness when processed images were compared (P, I, and P + I). Although most first-order, shape, and texture features showed good robustness, GLSZM small-area emphasis-related features and NGTDM strength were sensitive to variations in pixel size and slice thickness.

**Conclusion:**

Most MR radiomic features in patients with cervical cancer were robust after pixel size resampling and interpolation following the feature standardization process. The understanding regarding the robustness of individual features after pixel size resampling and interpolation could help future radiomics research.

**Supplementary Information:**

The online version contains supplementary material available at 10.1186/s40644-021-00388-5.

## Background

Radiomics is a promising field using quantitative image features extracted from medical imaging. The analysis of high-throughput data from various medical images, such as computed tomography (CT), magnetic resonance (MR), and positron emission tomography (PET), has become feasible using advanced computational power. Although radiomics can be used in various diseases, it has been the most used investigation tool in oncology. Radiomics could provide novel image biomarkers that could help cancer detection, diagnosis, assessment, and prediction of treatment response and prognosis [1].

However, the implementation of radiomics in clinical practice may be challenging. A major obstacle to its clinical application is that the robustness of extracted radiomic features is unclear. To establish novel quantitative imaging biomarkers in clinical practice, assessing feature robustness must be preceded. Recently, many researchers have focused on obtaining a more thorough understanding of feature characteristics and robustness [2–9]. However, most studies have used phantoms; thus, it is difficult to ensure that their results could be applied to imaging datasets of real patients [3–10].

Furthermore, it is difficult to standardize the parameters during image acquisition for all patients in clinical settings. For researchers trying to retrospectively investigate the datasets of real patients, sometimes, it is inevitable to analyze images acquired from various imaging acquisition protocols and scanners. Therefore, we often face situations where pixel size and slice thickness vary among patients. In this situation, pixel size resampling and interpolation should be used to standardize variable pixel sizes and slice thicknesses, respectively, to ensure reproducibility. Several studies have reported that pixel size resampling and interpolation improved reproducibility in CT radiomic features [11, 12], suggesting that such preprocessing steps are necessary.

Although pixel size resampling and interpolation have been known to be a necessary preprocessing step in radiomics research, the impact of slice thickness and pixel size on radiomic features has not been well understood. Although two phantom studies have reported the robustness of MR radiomic features recently [13, 14], studies on the robustness of radiomic features have focused primarily on CT and PET datasets [11, 15–17]. Moreover, to our knowledge, no studies have analyzed the impact of pixel size resampling and interpolation on the robustness of MR radiomic features of real patients’ dataset. Therefore, we hypothesize that pixel size resampling and interpolation significantly affect radiomic features. To test this hypothesis, we assessed the robustness of MR radiomic features to pixel size resampling and interpolation in patients with locally advanced cervical cancer.

## Methods

### Cervical Cancer MR image dataset

We included 254 patients who were pathologically diagnosed with stage IB-IVA cervical cancer and received definitive chemoradiation at our institution between January 2006 and June 2020. The characteristics of the patients are summarized in Table 1. Pretreatment MR scans were analyzed.

**Table 1:** Patient and tumor characteristics

Characteristic	*N* (%)
	Total *N* = 254
**Age (years)**	
median (range)	57 (23–86)
**Pathology**	
SCC	232 (91.34%)
Adenocarcinoma	15 (5.91%)
Adenosquamous carcinoma	7 (2.76%)
**FIGO stage**	
IB	3 (1.18%)
IIA	5 (1.97%)
IIB	200 (78.74%)
IIIA	11 (4.33%)
IIIB	25 (9.84%)
IVA	10 (3.94%)
**Extent of lymph node involvement**	
None	98 (38.58%)
Pelvic only	125 (49.21%)
Pelvic + para-aortic	31 (12.20%)
**Primary tumor size (mm)**	
median (range)	47 (14–110)

Abbreviations: *SCC* squamous cell carcinoma, *FIGO* International Federation of Gynecology and Obstetrics

Three MR scanners were used for MR acquisition (Discovery MR750, GE Healthcare; Magnetom Avanto, Siemens Healthcare; and Signa Excite, GE Healthcare) (see Additional file 1). A pelvic array coil for pelvic scans was used. Although the MR protocols varied in each patient, we obtained axial T1-weighted fast spin-echo (FSE) images after the administration of gadoliamide (T1E) from 252 patients and axial T2-weighted FSE images from 254 patients. The median slice thicknesses were 5.0 mm (range, 1.5–10 mm) and 5.3 mm (range, 3–10 mm) in T1E and T2 scans, respectively. The median matrix sizes were 336 (range, 208–720) in row and 448 (range, 232–720) in column. The median pixel space was 0.8 mm (range, 0.4–1.2 mm).

### Segmentation and feature extraction

Each cervical cancer region was semimanually segmented on the axial gadolinium-enhanced T1-weighted and T2-weighted images by two radiation oncologists (S.H. and B.B.). Segmentation was performed using the Eclipse treatment planning system, version 13.7 (Varian Medical Systems, Palo Alto, CA, USA). Each region of interest (ROI) was saved as voxels. Using this ROI as a 3-dimensional mask, radiomic features were extracted using Pyradiomics version 3.0 [18]. In this study, 18 first-order, 4 shape, 24 Gray-level co-occurrence matrix (GLCM), 16 Gray-level size zone matrix (GLSZM), 16 Gray-level run length matrix (GLRLM), 5 neighboring gray tone difference matrix (NGTDM), and 14 Gray-level dependence matrix (GLDM) features were extracted. All mathematical definitions and feature descriptions are available at https://pyradiomics.readthedocs.io/en/latest/. A fixed bin number of 64 was used for all analyses. In image processing and feature calculation, we followed the guidelines of the Image Biomarkers Standardization Initiative [2], and the image processing parameters are summarized in Table 2.

**Table 2:** Imaging processing parameters

Parameter	
**Image pixel space (mm)**	0.2, 0.4, 0.6, 0.8, 1
**Image slice thickness (mm)**	1, 3, 5, 7
**Image interpolation method**	Cubic spline algorithm
**ROI interpolation method**	Cubic spline algorithm
**Intensity normalization**	None, (x − mean(x))/SD(x)
**Discretisation method**	
Fixed bin number	64
**Image filter**	None

### Pixel space resampling and interpolation

The MR scans of the patients were interpolated and resampled using various slice thicknesses and pixel spaces to assess the effect of pixel space resampling and interpolation. Interpolation process translates image intensities from the original grid to a new grid. Several interpolation algorithms are used for interpolation, such as nearest neighbor, linear, and cubic spline interpolation. Nearest neighbor is a zero-order polynomial method that signs gray-level values of the nearest neighbor to the interpolated point. In 3-dimensional calculation, linear interpolation uses the intensities of the eight nearby voxels in the original grid to calculate a new intensity using linear interpolation. Cubic spline interpolation uses a larger neighborhood to generate a continuous third-order polynomial at the voxel centers in the new grid. Hence, cubic spline interpolation can have smoother surface than linear methods, while being slower in implementation [19]. Compared with linear interpolation that acts as a low-pass filter, cubic spline interpolation tends to preserve high-frequency content more in upsampling circumstances [20, 21]. Because our analysis included an upsampling process as well as a downsampling process, we used the cubic spline algorithm to interpolate the MR scans.

**Fig. 1:**
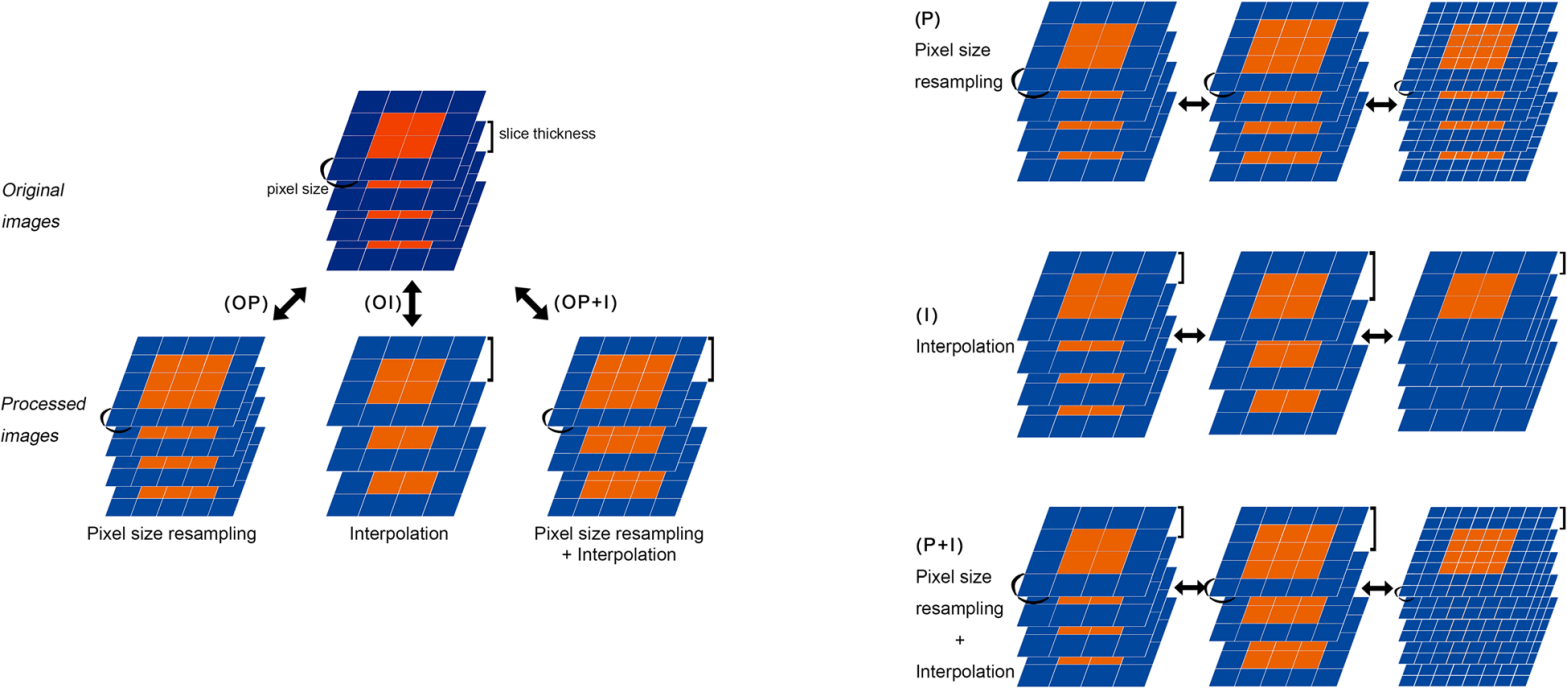
Workflow to derive the intraclass correlation coefficients (ICCs). ICC was calculated between the original images and the images that underwent pixel size resampling of 0.6 mm (OP), the original images and the images that underwent interpolation (slice thickness: 5 mm) (OI), and the original images and the images that underwent resampling and interpolation of pixel size (OP+I), pixel size-resampled image sets (P), interpolated image sets (I), and pixel space-resampled and interpolated image sets (P + I)

Figure 1 shows the schematic diagram of the experimental design. First, we measured the variability in three experimental groups to investigate the concordance between the original data (no pixel size resampling or interpolation) and processed data. Intraclass correlation coefficient (ICC) was calculated between the original images and the images that underwent pixel size resampling of 0.6 mm (OP), the original images and the images that underwent interpolation (slice thickness: 5 mm) (OI), and the original images and the images that underwent pixel size resampling and interpolation (OP+I) were investigated. Second, we measured the concordance among processed image sets to assess which process affects feature robustness: pixel spaces of 0.2 mm, 0.4 mm, 0.6 mm, 0.8 mm, and 1 mm (P); slice thicknesses of 1 mm, 3 mm, 5 mm, and 7 mm (I); and pixel spaces and slice thicknesses of 0.2 mm and 1 mm, 0.4 mm and 3 mm, 0.6 mm and 5 mm, 0.8 mm and 7 mm, and 1 mm and 10 mm, respectively (PI) (Fig. 1).

### Intensity normalization

MR signal intensity normalization (IN) was performed using Pyradiomics. Pyradiomics enabled the normalization of image intensity values. Normalization centered the image at the mean with standard deviation (SD) [22, 23]. Normalization was based on all the gray values contained within the image and not just those defined by ROI.

$$ \mathrm{f}\left(\mathrm{x}\right)=\frac{s\left(x-{\mu}_x\right)}{\sigma_x}, $$

where x and f(x) represent the original and normalized intensity, respectively, *μ*_*x*_ and *σ*_*x*_ represent the mean and SD of the image intensity values, respectively, and s is a scaling factor, which was set to 100. All voxels values were shifted by 300 to ensure that the majority of voxels had positive values.

### Feature standardization

Each feature was standardized using z-score normalization [z = (x − mean(x))/SD(x)] so that each feature has a same mean of 0 and a standard deviation of 1, contributing to the standard normal distribution [24]. We tested the robustness with and without the feature standardization process.

### Statistical analysis

ICC was used to assess feature robustness to pixel size resampling, interpolation, and both [25]. ICC was defined as follows:

$$ \mathrm{ICC}=\frac{MS_R-{MS}_E}{MS_R+\left(k-1\right){MS}_E+\frac{k}{n}\left({MS}_C-{MS}_E\right)}, $$

where MS_R_ represents the mean square for feature values, MS_E_ represents the mean square for error, MS_C_ represents the mean square for repeated measures, k represents the number of repeated acquisitions, and n represents the number of patients. ICC has been used to measure the reproducibility and reliability of numeric measurements organized into groups [26–29]. It has the advantage of being able to compare more than two groups of variables. Although ICC has a limitation for comparing reproducibility in different populations, our analysis did not include comparisons in different populations. The features having ICC values of < 0.5, 0.5–0.84, and ≥ 0.85 were categorized as poor, fair, and good robustness, respectively. All statistical analyses were performed using R (ver. 3.6.3; The R Foundation, Indianapolis, IN, USA).

## Results

### Comparison between the original and processed scans

**Fig. 2:**
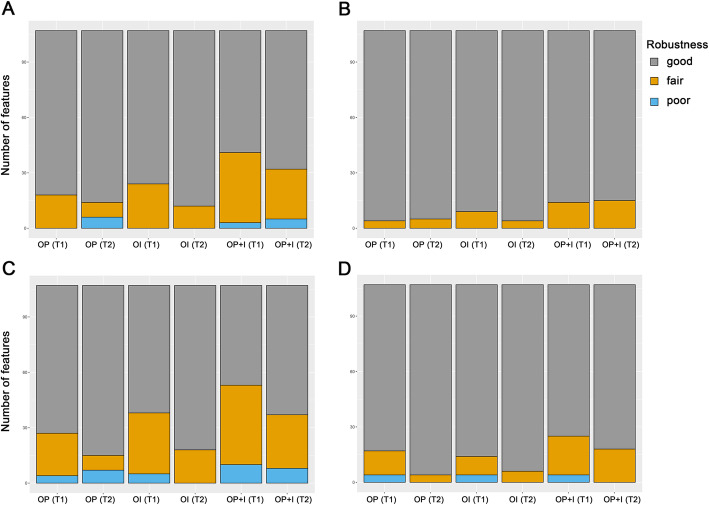
Proportions of features having good (gray), fair (yellow), and poor robustness (blue). Intraclass correlation coefficient (ICC) values between the original images and the images that underwent pixel size resampling of 0.6 mm (OP), the original images and the images that underwent interpolation (slice thickness: 5 mm) (OI), and the original images and the images that underwent pixel size resampling and interpolation (OP+I) were evaluated. **a** Original images without feature standardization. **b** Original images with feature standardization. **c** Intensity-normalized images without feature standardization. **d** Intensity-normalized images with feature standardization

The proportions of the features having good, fair, and poor robustness are shown in Fig. 2. In the non-IN images, 83.2%/86.9, 77.6%/88.8, and 61.7%/70.1% of the T1E/T2 features showed good robustness in the OP, OI, and OP+I comparison groups, respectively (Fig. 2a). The proportions of features showing fair robustness in the OP, OI, and OP+I comparison groups were 16.8%/7.5, 22.4%/11.2, and 35.5%/25.2% in the T1−/T2-weighted images, respectively. The proportions of features exhibiting poor robustness in the OP, OI, and OP+I comparison groups were 0.0%/5.6, 0.0%/0.0, and 2.8%/4.7% in the T1−/T2-weighted images, respectively. In the IN images, 74.8%/86.0, 64.5%/83.2%, and 50.5/65.4% of the T1E/T2 features showed good robustness in the OP, OI, and OP+I comparison groups, respectively (Fig. 2c). The proportions of features exhibiting fair robustness in the OP, OI, and OP+I comparison groups were 21.5%/7.5, 30.8%/16.8, and 40.2%/27.1% in the T1−/T2-weighted images, respectively. The proportions of features showing poor robustness in the OP, OI, and OP+I comparison groups were 3.7%/6.5, 4.7%/0.0, and 9.3%/7.5% in the T1−/T2-weighted images, respectively.

Each feature was standardized using z-score standardization. After the feature standardization process, the proportion of features having good robustness increased in both the non-IN and IN images (Fig. 2b and d, respectively). In the non-IN images with feature standardization, 96.3%/95.3, 91.6%/96.3, and 86.9%/86.0% of the T1E/T2 features showed good robustness in the OP, OI, and OP+I comparison groups, respectively (Fig. 2b). The proportions of features showing fair robustness in the OP, OI, and OP+I comparison groups were 3.7%/4.7, 8.4%/3.7, and 13.1%/14.0% in the T1−/T2-weighted images, respectively. None of the features showed poor robustness after feature standardization in the non-IN images. In the IN images with feature standardization, 84.1%/96.3, 86.9%/94.4, and 76.6%/83.2% of the T1E/T2 features showed good robustness in the OP, OI, and OP+I comparison groups, respectively (Fig. 2d). The proportions of features showing fair robustness in the OP, OI, and OP+I comparison groups were 12.1%/3.7, 9.3%/5.6, and 19.6%/16.8% in the T1−/T2-weighted images, respectively. The proportions of features exhibiting poor robustness in the OP, OI, and OP+I comparison groups were 3.7%/0.0, 3.7%/0.0, and 3.7%/0.0% in the T1−/T2-weighted images, respectively.

**Fig. 3:**
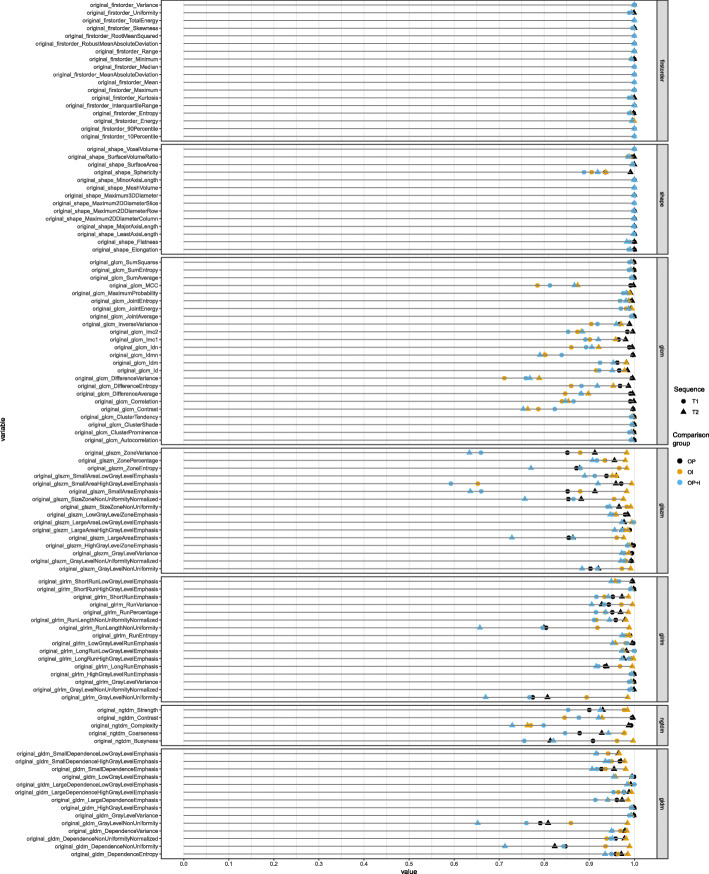
Robustness analysis of 107 radiomic features to resampling and interpolation of pixel size from original images after feature standardization. The colors represent the images that have been compared (black: original images and images that underwent pixel size resampling; yellow: original images and images that underwent interpolation; blue: original images and images that underwent pixel size resampling and interpolation). The shapes represent the sequence of magnetic resonance images (circle: T1-weighted images; triangle: T2-weighted images)

**Fig. 4:**
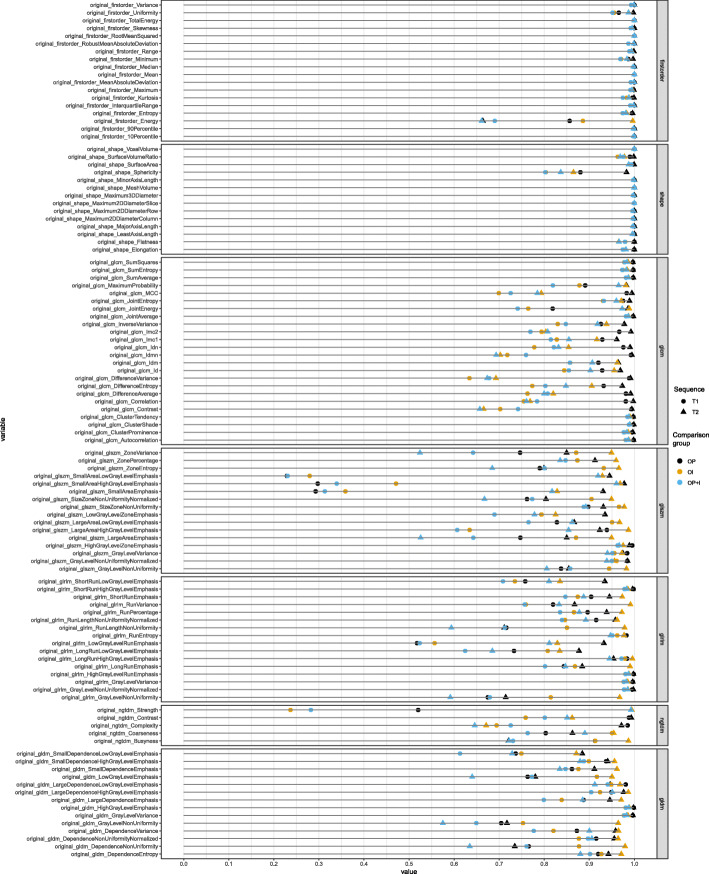
Robustness analysis of 107 radiomic features to resampling and interpolation of pixel size from intensity-normalized images after feature standardization. The colors represent the images that have been compared (black: original images and images that underwent pixel size resampling; yellow: original images and images that underwent interpolation; blue: original images and images that underwent pixel size resampling and interpolation). The shapes represent the sequence of magnetic resonance images (circle: T1-weighted image; triangle: T2-weighted images)

Figures 3 and 4 show the ICC values of individual features from the non-IN and IN images, respectively. First-order, shape, and GLCM features were robust to the pixel size resampling and interpolation processes. Poor robustness was found in small area-related features of GLSZM and strength of NGTDM of IN images, whereas these features showed good or fair robustness in the non-IN images.

### Comparison among processed scans

**Fig. 5:**
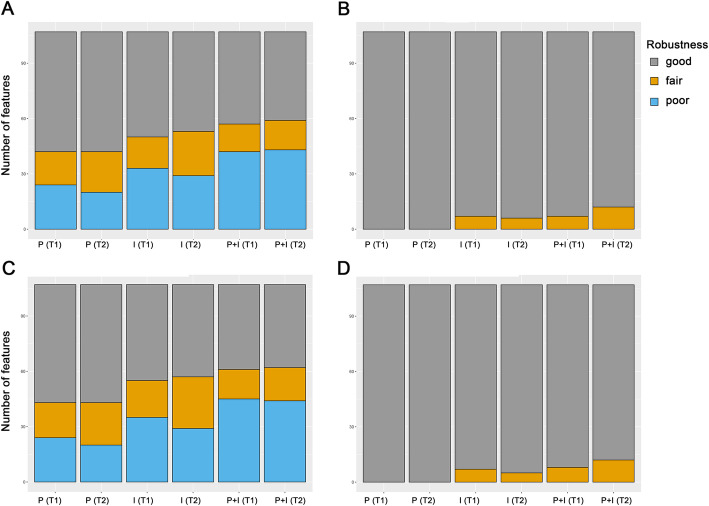
Proportions of features having good (gray), fair (yellow), and poor robustness (blue). Intraclass correlation coefficient (ICC) values among pixel size resampling (P), interpolation (I), resampling and interpolation of pixel size (P + I) images were evaluated. **a** Original images without feature standardization. **b** Original images with feature standardization. **c** Intensity-normalized images without feature standardization. **d** Intensity-normalized images with feature standardization

In the non-IN images, 60.7%/60.7, 53.3%/50.5, and 46.7%/44.9% of the features showed good robustness in the P, I, and P + I comparison groups, respectively (Fig. 5a). The proportions of features exhibiting fair robustness in the P, I, and P + I comparison groups were 16.8%/20.6, 15.9%/22.4, and 14.0%/15.0% in the T1−/T2-weighted images, respectively. The proportions of features showing poor robustness in the P, I, and P + I comparison groups were 22.4%/18.7, 30.8%/27.1, and 39.3%/40.2% in the T1−/T2-weighted images, respectively. In the IN images, 59.8%/59.8, 48.6%/46.7, and 43.0%/42.1% showed good robustness in the P, I, and P + I comparison groups, respectively (Fig. 5c). The proportions of features showing fair robustness in the P, I, and P + I comparison groups were 17.8%/21.5, 18.7%/26.2, and 15.0%/16.8% in the T1−/T2-weighted images, respectively. The proportions of features exhibiting poor robustness in the P, I, and P + I comparison groups were 22.4%/18.7, 32.7%/27.1, and 42.1%/41.1% in the T1−/T2-weighted images, respectively.

The proportions of features with good, fair, and poor repeatability after feature standardization are depicted in Fig. 5b and d. In general, most features (≥88.8%) showed good robustness after feature standardization. Specifically, in the non-IN images after feature standardization, 100.0%/100.0, 93.5%/94.4, and 93.5%/88.8% of the T1E/T2 features showed good robustness in the P, I, and P + I comparison groups, respectively (Fig. 5b). The proportions of features showing fair robustness in the P, I, and P + I comparison groups were 0.0%/0.0, 6.5%/5.6, and 6.5%/11.2% in the T1−/T2-weighted images, respectively. Similar results were found in the IN images after feature standardization, with 100.0%/100.0, 93.5%/92.5, and 92.5%/88.8% of T1E/T2 features showing good robustness in the P, I, and P + I comparison groups, respectively (Fig. 5d). The proportions of features showing fair robustness in the P, I, and P + I comparison groups were 0.0%/0.0, 6.5%/4.7, and 7.5%/11.2% in the T1−/T2-weighted images, respectively. None of the features showed poor robustness after feature standardization.

The features from interpolated scans were less consistent compared with those from pixel size-resampled scans, suggesting that features can be more affected by slice thickness interpolation than pixel size resampling process. The proportion of features having good robustness was lowest in images that underwent the pixel size resampling and interpolation process (Fig. 5).

Regarding the ICC values of individual feature, all features in the first-order and shape categories showed good robustness to various pixel sizes and slice thicknesses. In the non-IN images, all features in the first-order, shape, GLSZM, GLRLM, NGTDM, and GLDM categories had good robustness after feature standardization (see Additional file 2). Although 12.5% (3 of 107) of the features showed fair robustness, all features showed ICC values of > 0.7. In the analysis of IN images, small-area emphasis-related GLSZM and NGTDM strength features showed poor to fair robustness (see Additional file 3).

### Intensity normalization

**Fig. 6:**
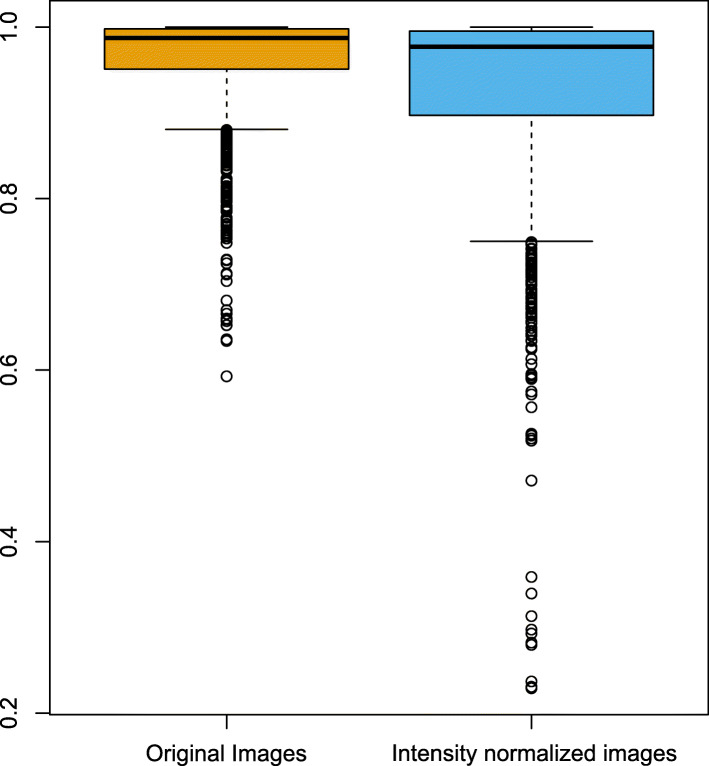
The intraclass correlation coefficients (ICCs) of non-intensity normalized images (yellow) and intensity-normalized images (blue). The ICC values were significantly lower in intensity-normalized images, suggesting that the features from intensity-normalized images were less consistent than those from non-intensity normalized images

We compared the ICC values in terms of IN from the comparison between the original and processed scans (Fig. 3b and d) and between the processed scans after feature standardization (Fig. 5b and d; Fig. 6). The ICC values were significantly lower in the IN images than in the non-IN images (*p* < 0.001), indicating that features from IN images were less consistent. Note that NGTDM strength features were sensitive only in IN images, whereas they were robust in non-IN images (Figs. 3 and 4) (see Additional files 2 and 3).

## Discussion

In this study, we found that MR radiomic features tended to be robust to pixel size resampling, interpolation, and both (Fig. 1). Especially, most first-order and shape features showed excellent concordance after pixel size resampling and interpolation, suggesting that they were not sensitive to pixel space resampling or interpolation. Notably, feature standardization process could improve robustness in all comparison groups.

Although most features were consistent with various pixel spaces and slice thicknesses (Figs. 3 and 4), GLSZM small-area emphasis-related features and NGTDM strength were not consistent to pixel size resampling and interpolation. GLSZM small-area emphasis is a measure of the distribution of small-size zones. When it has smaller size zones and more fine textures, the value of GLSZM small-area emphasis is high. Because pixel size resampling and interpolation transform the original image to some extent, small volume-related features could be more affected by them rather than large volumes. In case of data with various pixel sizes and slice thicknesses are analyzed, for example, in our dataset, a researcher might need to exclude small volume-related features because their robustness to pixel size resampling and interpolation is uncertain.

Studies on the effect of variations in pixel size and slice thickness on radiomic features were mainly using CT and PET datasets [30–33]. In most studies reported thus far, radiomic features were sensitive to variations in pixel size and slice thickness [30–33]. In a phantom study conducted by Zhao et al., the slice thickness and reconstruction algorithm significantly affected the CT radiomic features [32]. More recently, the same group conducted a CT study on patients with lung cancer. They reported that resampling CT images using different slice thicknesses and reconstruction kernels resulted in a low reproducibility in CT radiomic features [31]. Similarly, Shafig-ul-Hassan demonstrated the dependency on voxel size and gray level of CT radiomic features in their phantom study [11]. Consistent with the CT studies, in a study assessing the robustness against interpolation in 18F-fluorodeoxyglucose PET images for 441 patients with esophageal cancer, only 66.0% of PET radiomic features were robust to the interpolation [33]. However, our findings seem to contrast with those from the aforementioned studies [30–33]. In our study, > 80% of features were consistent in all comparison groups. The plausible reasons for this discrepant finding were that simple z-score standardization of each feature increased robustness and that MR intensity was relative values in nature so that they are more robust to pixel size or slice thickness than CT and PET values. Another explanation may be that our MR datasets were acquired using various scanners and scanning protocols; therefore, our datasets could obtain generalizability by itself over variations in pixel size or slice thickness.

More recently, two studies have investigated the robustness of MR radiomic features [13, 14]. Baeßler et al. performed the test–retest analysis as well as intraobserver and interobserver analysis using multiple MR sequences [14]. They reported that the number of robust features was higher for features (81%) from FLAIR than for features from T1- and T2-weighted images. In their report, 33% of the features showed excellent robustness across all sequences and excellent intraobserver and interobserver reproducibility. Bianchini et al. also evaluated the robustness of MR radiomic features in various scenarios. They tested the robustness with phantom repositioning, different scanners, and different acquisition parameters such as echo time and pulse repetition time. Consistent with our results, > 80% of the features showed excellent reproducibility.

In addition to variations in pixel size and slice thickness, MR radiomic analysis suffers from a wide variability in pixel intensity resulting from using various scanners, manufacturers, and acquisition parameters. To deal with nonstandardized MR intensity, the IN process could make MR radiomics more reliable. Several studies have demonstrated that the IN process was a necessary step for analyzing MR image features [22, 23, 34]. In contrast to a study by Carre et al., in which the IN process improved the robustness of first-order and second-order features [23], our study showed decreased robustness in IN images compared with non-IN images to pixel size resampling and interpolation (Figs. 2, 3, 4, 5 and 6). A possible explanation is that the IN process might transform the original image and lose the original information partly, even though it could mitigate the influence of various MRI acquisition protocols. By doing so, the reproducibility of MR features against pixel size resampling and interpolation process might be decreased for IN images. Our results highlight the need for caution when applying the processes of pixel size resampling and interpolation in IN images. For example, NGTDM strength features showed good concordance in the non-IN images, whereas they had fair to poor concordance in the IN images (Figs. 3 and 4) (see Additional files 2 and 3).

Despite the encouraging results, our study has several limitations. First, our results could be specific to our dataset. It might be difficult to apply the results to other datasets. Second, due to its retrospective design, the MR scanners and scanning protocols used varied. However, various scanning protocols including pixel size and slice thickness of the original scans might have a positive influence on model generalizability built from these features. Third, we did not analyze the effect of other preprocessing methods such as filtering and gray-level discretization. Nevertheless, our study provides important information on the robustness of MR radiomic features to pixel size resampling and interpolation. Therefore, our results, in which the robustness of MR features increased following simple z-score standardization, would provide information that could help future MR radiomic studies.

## Conclusion

Most of the MR radiomic features in patients with cervical cancer were robust with respect to pixel size resampling and interpolation. The feature standardization process could improve the robustness. Most first-order, shape, GLCM, GLRLM, and GLDM features showed good robustness. However, GLSZM small-area emphasis-related features and NGTDM strength was not consistent in the IN images. MR features might be more affected by slice thickness interpolation than pixel size resampling process. The understanding regarding the robustness of individual features after pixel size resampling and interpolation could help future radiomics research.

## Supplementary Information

**Additional file 1.** List of MR scanners and their manufacturers.

**Additional file 2.** Robustness analysis of 107 radiomic features to pixel size resampling and interpolation from original images after feature standardization. The colors represent the images that have been compared (black: pixel size resampling images; yellow: interpolation images; blue: pixel size resampling and interpolation images). The shapes represent the sequence of magnetic resonance images (circle: T1-weighted images; triangle: T2-weighted images).

**Additional file 3.** Robustness analysis of 107 radiomic features to pixel size resampling and interpolation from intensity-normalized images after feature standardization. The colors represent the images that have been compared (black: pixel size resampling images; yellow: interpolation images; blue: pixel size resampling and interpolation images). The shapes represent the sequence of magnetic resonance images (circle: T1-weighted images; triangle: T2-weighted images).

## Data Availability

The datasets generated and/or analyzed during the current study are not publicly available due to the privacy protection policy of personal medical information of our institution but are available from the corresponding author on reasonable request.

## Abbreviations

GLCM: Gray-level co-occurrence matrix
GLDM: Gray-level dependence matrix
GLSZM: Gray-level size zone matrix
GLRLM: Gray-level run length matrix
NGTDM: Neighboring gray tone difference matrix
ICC: Intraclass correlation coefficient
OP: Original images and images that underwent pixel size resampling
OI: Original images and images that underwent interpolation
OP+I: Original images and images that underwent resampling and interpolation of pixel size
P: Processed image sets with various pixel spaces
I: Processed image sets with various slice thicknesses
P + I: Processed image sets with various pixel sizes and slice thicknesses
IN: Intensity normalization
