# Supplementary material for: Robustness of magnetic resonance radiomic features to pixel size resampling and interpolation in patients with cervical cancer
Source: Cancer Imaging. 2021 Feb 2;21:19. doi: 10.1186/s40644-021-00388-5 (PMC7856733; doi:10.1186/s40644-021-00388-5)
Supplement: Supplementary file 1 — Additional file 1. List of MR scanners and their manufacturers. [file 40644_2021_388_MOESM1_ESM.docx]

# Additional File 1.

| List of MR scanners and their manufacturers. | | | |
| --- | --- | --- | --- |
| **Model** | **Manufacturer** | **Tesla (T)** | **Patient no. (%)** |
| Magnetom Vision plus | Siemens | 3.0 | 126 (49.5) |
| Avanto | Siemens | 1.5 | 76 (30.1) |
| Discovery MR750 | GE Medical Systems | 1.5 | 1. 20.4) |
