# Supplementary figures and images for: Robustness of magnetic resonance radiomic features to pixel size resampling and interpolation in patients with cervical cancer
Source: Cancer Imaging. 2021 Feb 2;21:19. doi: 10.1186/s40644-021-00388-5 (PMC7856733; doi:10.1186/s40644-021-00388-5)

variable

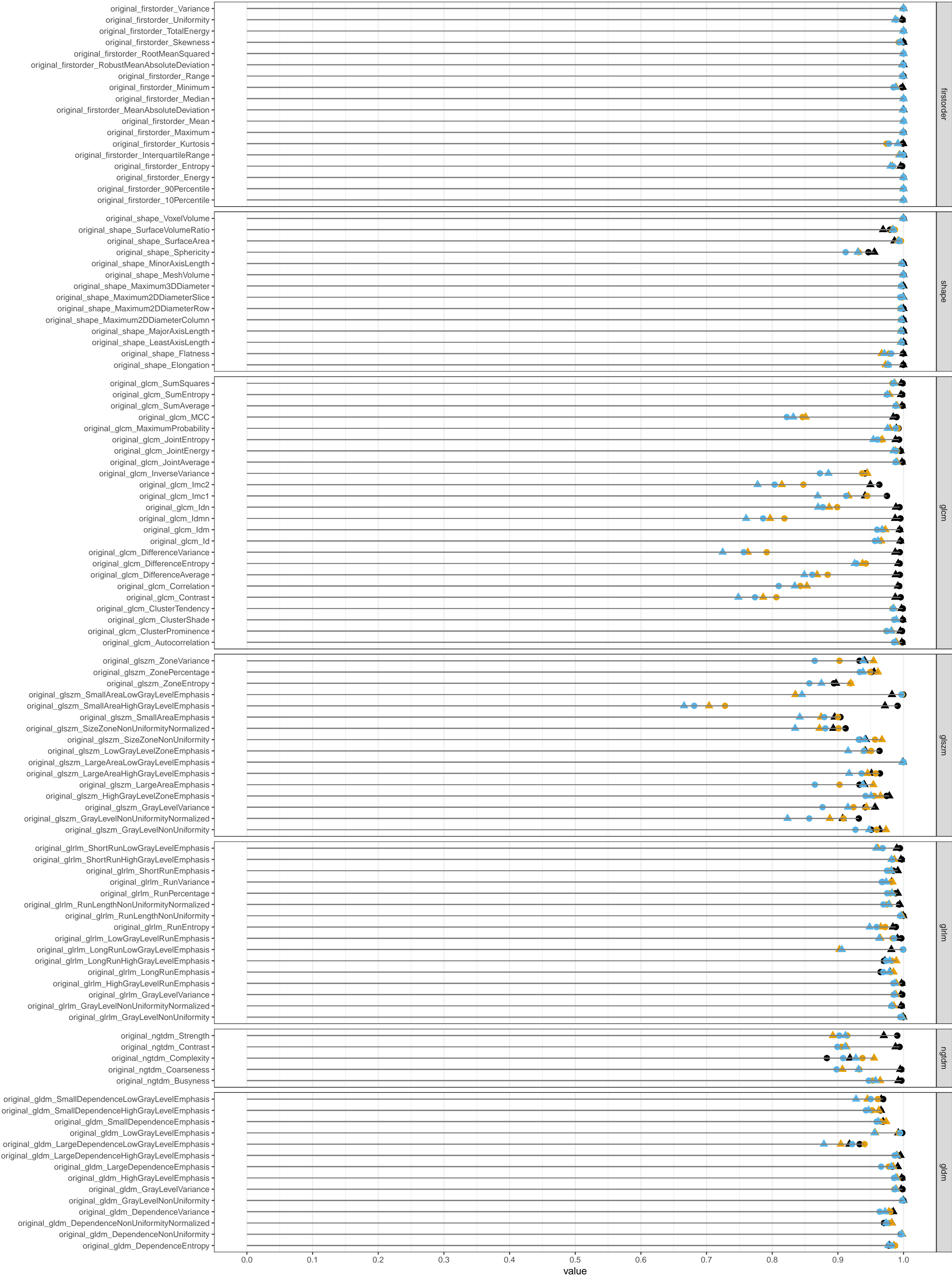

Sequence  
● T1  
▲ T2

Comparison group  
● P  
● I  
● P+I

Supplement: Supplementary file 2 — Additional file 2. Robustness analysis of 107 radiomic features to pixel size resampling and interpolation from original images after feature standardization. The colors represent the images that have been compared (black: pixel size resampling images; yellow: interpolation images; blue: pixel size resampling and interpolation images). The shapes represent the sequence of magnetic resonance images (circle: T1-weighted images; triangle: T2-weighted images). [file 40644_2021_388_MOESM2_ESM.pdf]

variable

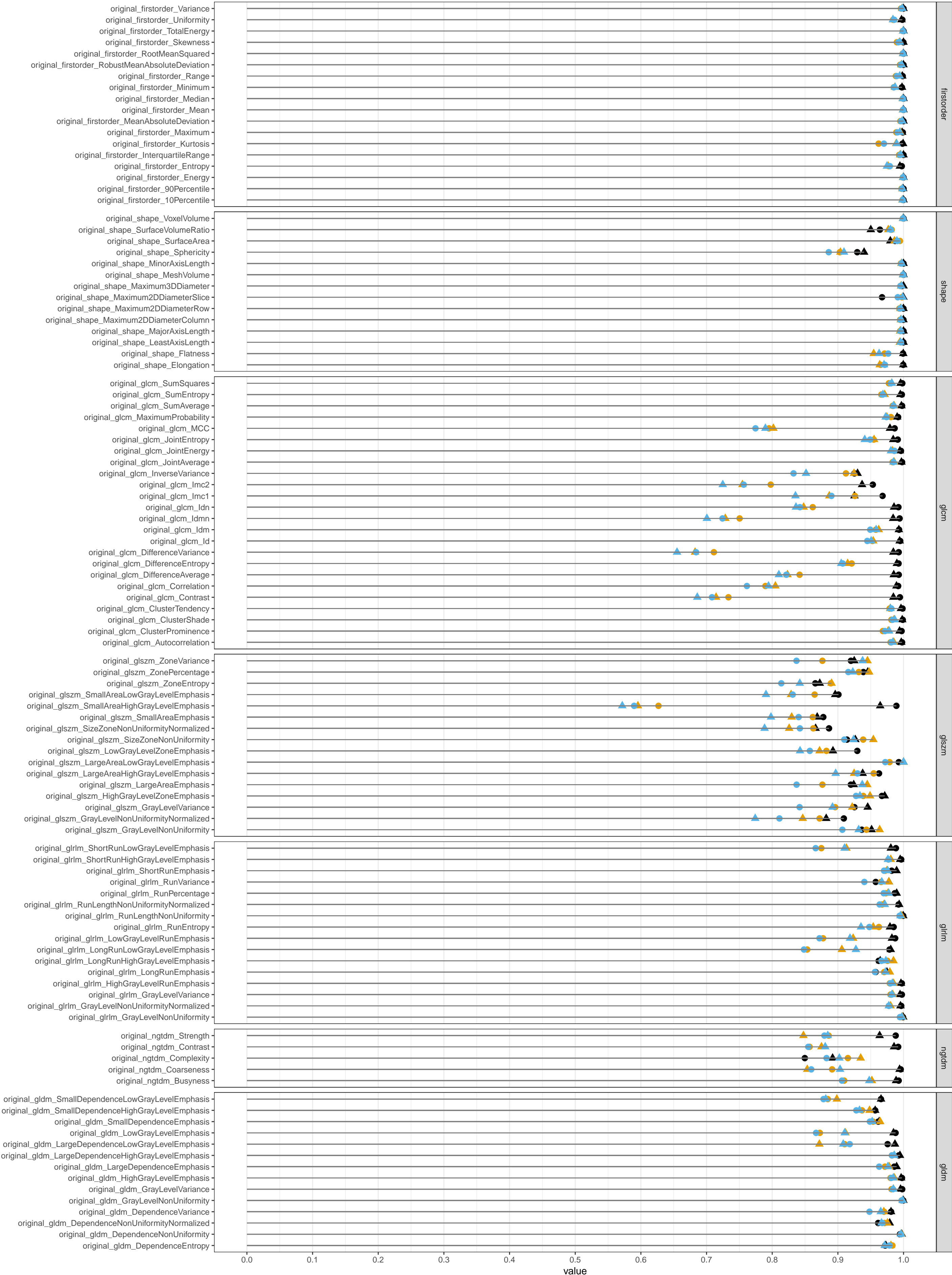

Supplement: Supplementary file 3 — Additional file 3. Robustness analysis of 107 radiomic features to pixel size resampling and interpolation from intensity-normalized images after feature standardization. The colors represent the images that have been compared (black: pixel size resampling images; yellow: interpolation images; blue: pixel size resampling and interpolation images). The shapes represent the sequence of magnetic resonance images (circle: T1-weighted images; triangle: T2-weighted images). [file 40644_2021_388_MOESM3_ESM.pdf]
